# Study protocol for the DISTINCT trial: inDividual, targeted thrombosIS prophylaxis versus the standard ‘one-size-fits-all’ approach in patients undergoing Total hIp or total kNee replaCemenT – a national, multicentre, randomised, multiarm, open-label trial

**DOI:** 10.1136/bmjopen-2025-101180

**Published:** 2025-10-06

**Authors:** Ruben Y Kok, Leti van Bodegom-Vos, Harmen B Ettema, Rolf H H Groenwold, Wilbert B van den Hout, Menno V Huisman, Frederikus A Klok, Rob G H H Nelissen, Nienke van Rein, Merel van Veen, Stephan B W Vehmeijer, Jan Joost I Wiegerinck, Suzanne C Cannegieter, Banne Nemeth

**Affiliations:** 1 Department of Clinical Epidemiology, Leiden University Medical Center, Leiden, The Netherlands; 2 Department of Biomedical Data Sciences, Leiden University Medical Center, Leiden, The Netherlands; 3 Department of Orthopedics, Isala Klinieken, Zwolle, The Netherlands; 4 Department of Medicine - Thrombosis and Haemostasis, Leiden University Medical Center, Leiden, The Netherlands; 5 Department of Orthopaedics, Leiden University Medical Center, Leiden, The Netherlands; 6 Department of Clinical Pharmacy and Toxicology, Leiden University Medical Center, Leiden, The Netherlands; 7 Dutch Hip Patient association 'Vereniging Afwijkende Heupontwikkeling (VAH)’, Nijkerk, The Netherlands; 8 Department of Orthopedics, Reinier Haga Orthopedisch Centrum, Zoetermeer, The Netherlands; 9 Department of Orthopedics, Bergman Clinics Musculoskeletal Division, Naarden, The Netherlands

**Keywords:** Orthopedics, Thromboembolism, Hip, Knee

## Abstract

**Introduction:**

Patients undergoing total hip arthroplasty (THA) and total knee arthroplasty (TKA) are considered to have a symptomatic venous thromboembolism (VTE) risk of 1.0%–1.5% despite thromboprophylaxis. Fast-track treatment protocols have substantially lowered the VTE risk in most patients. Hence, the majority of patients may be unnecessarily exposed to the burden and risk of thromboprophylaxis. On the contrary, there are still patients with a high VTE risk who develop VTE despite thromboprophylaxis. Thus, tailored thromboprophylaxis treatment may potentially reduce both VTE and bleeding risk.

**Methods and analysis:**

The DISTINCT (inDividual, targeted thrombosIS prophylaxis versus the standard ‘one-size-fits-all’ approach in patients undergoing Total hIp or total kNee replaCemenT) trial is a national, multicentre, randomised, multiarm, open-label trial. The main objective is to study whether tailored thromboprophylaxis reduces the occurrence of symptomatic VTE (primary outcome) and major bleeding (primary safety outcome) within 90 days after THA/TKA in comparison with standard thromboprophylaxis. Patients with a low, intermediate or high predicted VTE risk (based on the Thrombosis Risk Prediction following total hip and knee arthroplasty score (TRiP(plasty) score)) will be included in the DISTINCT-1, DISTINCT-2 or DISTINCT-3 studies, respectively. In the DISTINCT-1 trial, 3478 patients will be randomly allocated to receive either in-hospital thromboprophylaxis or standard prophylaxis. In the DISTINCT-2 cohort study, 2500 patients will receive standard prophylaxis. In the DISTINCT-3 trial, 4100 patients will be randomly allocated to receive either 6 weeks of high-dose thromboprophylaxis or standard prophylaxis. Standard prophylaxis consists of a low dose of any approved thromboprophylactic agent for 4 weeks. We hypothesise that (1) the efficacy of in-hospital only thromboprophylaxis is non-inferior in preventing VTE and equally safe compared with standard prophylaxis in patients with a low VTE risk (DISTINCT-1) and (2) prolonged high-dose thromboprophylaxis is superior in preventing VTE as compared with standard prophylaxis in patients with a high VTE risk (DISTINCT-3). Patients with intermediate VTE risk will be observed to evaluate VTE and bleeding rates (DISTINCT-2).

**Ethics and dissemination:**

The protocol has been approved by the Medical Research Ethics Committee Leiden-Den Haag-Delft, EU-trial-number 2023-510186-98. Study results will be disseminated through peer-reviewed journals and during international conferences.

**Trial registration number:**

NCT06581965.

**STRENGTHS AND LIMITATIONS OF THIS STUDY**

- The DISTINCT (inDividual, targeted thrombosIS prophylaxis versus the standard ‘one-size-fits-all’ approach in patients undergoing Total hIp or total kNee replaCemenT) trial is designed as a pragmatic trial to be integrated into routine clinical practice, allowing for the determination of an individual’s thromboprophylaxis regimen prior to surgery.
- The DISTINCT trial is the first thromboprophylaxis trial following arthroplasty that adapts treatment to an individual’s venous thromboembolism risk.
- Given that both minor and major surgery-related bleeding events following arthroplasty could lead to prosthetic joint infections (PJIs), the study includes PJI as a study outcome.
- The enrolment of a large number of patients from various teaching hospitals, general hospitals and private clinics across the Netherlands increases the generalisability of this study.
- The large sample size is both a recruitment challenge and a potential limitation of the feasibility of this study.

## Introduction

 Venous thromboembolism (VTE)^1^ primarily affects the deep veins of the leg (deep vein thrombosis (DVT)) and the pulmonary arteries (pulmonary embolism (PE)^2^). VTE is not only associated with considerable chronic morbidity leading to loss of quality of life but also with mortality. Patients undergoing elective total hip arthroplasty (THA) or total knee arthroplasty (TKA) have a high risk of postoperative VTE. Therefore, guidelines recommend prophylactic anticoagulant therapy after THA and TKA for all patients, although the type and length of treatment are under debate and differ between guidelines.^3^,^5^

Despite pharmacological thromboprophylaxis, 1.0%–1.5% of all patients still develop symptomatic VTE after THA/TKA.^6^ The main problem with the current ‘one-size-fits-all’ approach is that patients are considered to have an equal risk of thrombosis, while in reality, various individual and surgery-related factors influence VTE risk. For example, fast-track surgery protocols,^7^ which involve early mobilisation with subsequent reduction of hospital stay and opioid-sparing anaesthesia protocols, have greatly reduced VTE risk.^8^ Multiple studies have shown that in a large unselected population of patients undergoing THA/TKA, the incidence of VTE following fast-track surgery is <1.0% within 3 months, even when thromboprophylaxis was only administered during hospital admission (1–2 days).^8^,^11^ This implies that a large group of patients might currently be overtreated with thromboprophylactic therapy and are unnecessarily exposed to its burden and associated risk of bleeding. Major bleeding occurs in at least 0.5% of anticoagulated patients after THA/TKA, and minor bleeding, including clinically relevant non-major bleeding (CRNMB), occurs in about 6%.^12 13^ In addition to the general morbidity related to bleeding, a large surgery site-related haematoma increases the risk of an implant-associated infection and, therefore, the likelihood of revision surgery.^14^ Moreover, bleeding leads to prolonged wound drainage, which necessitates extended hospitalisation.^15^

At the other side of the spectrum, there must be patients with a high risk of VTE, as the average risk in the overall THA/TKA population is still 1.0%–1.5% despite thromboprophylaxis therapy.^6^ For instance, in patients with a history of VTE, the VTE risk following surgery is between 5% and 10%.^16^ For these patients, the current dose and duration of thromboprophylaxis might be insufficient, as they develop VTE despite prophylaxis.

Considering the above, important improvements can be expected from a tailored thromboprophylaxis strategy based on an individual’s risk profile. For patients with a low VTE risk, short-term thromboprophylaxis may be sufficient, while patients with a high VTE risk may benefit from an increased duration and higher, therapeutically dosed thromboprophylaxis. Hence, both thromboembolic and bleeding complications could be minimised, and a large proportion of patients will not need to be exposed to 4 weeks of anticoagulants, leading to societal cost savings.^17^

### Study objectives

The DISTINCT (inDividual, targeted thrombosIS prophylaxis versus the standard ‘one-size-fits-all’ approach in patients undergoing Total hIp or total kNee replaCemenT) trial aims to tailor thromboprophylaxis based on an individual’s estimated VTE risk. This VTE risk will be predicted by the recently developed and externally validated Thrombosis Risk Prediction following total hip and knee arthroplasty (TRiP(plasty)) score, specifically designed to predict an individual’s 90-day postoperative VTE risk after THA and TKA.^18^ We hypothesise that:

1. In patients with a low 90-day postoperative VTE risk (predicted risk <1.0%), thromboprophylaxis can be safely shortened to in-hospital duration only, without increasing the VTE risk, potentially reducing the incidence of CRNMB and minor bleeds.
2. In patients with a high 90-day postoperative VTE risk (predicted risk >1.5%), high-dose thromboprophylaxis for 6 weeks is more effective in preventing symptomatic VTE compared with the standard prophylaxis. In addition, we expect that the benefits of this approach outweigh the potentially increased risk of bleeding.

Patients with an intermediate 90-day postoperative VTE risk (predicted risk ≥1.0% and ≤1.5%) receiving standard prophylaxis will be included to validate the predicted VTE risk. In addition, this group acts as a safety margin to ensure patient safety for the abovementioned interventions.

## Methods and analysis

### Summary of project status

Medical Research Ethics Committee approval was obtained in September 2024, with the first patient enrolled in November 2024. Recruitment is currently underway in nine hospitals in the Netherlands, with more than 850 patients enrolled to date. Final enrolment is planned for March 2027 in DISTINCT-1, March 2028 in DISTINCT-2 and July 2030 in DISTINCT-3.

### Study design

The DISTINCT trial is a national, multicentre, randomised, multiarm, open-label trial with blinded outcome adjudication. Patients will be recruited from teaching hospitals, general hospitals and private clinics across the Netherlands.

**Figure 1:**
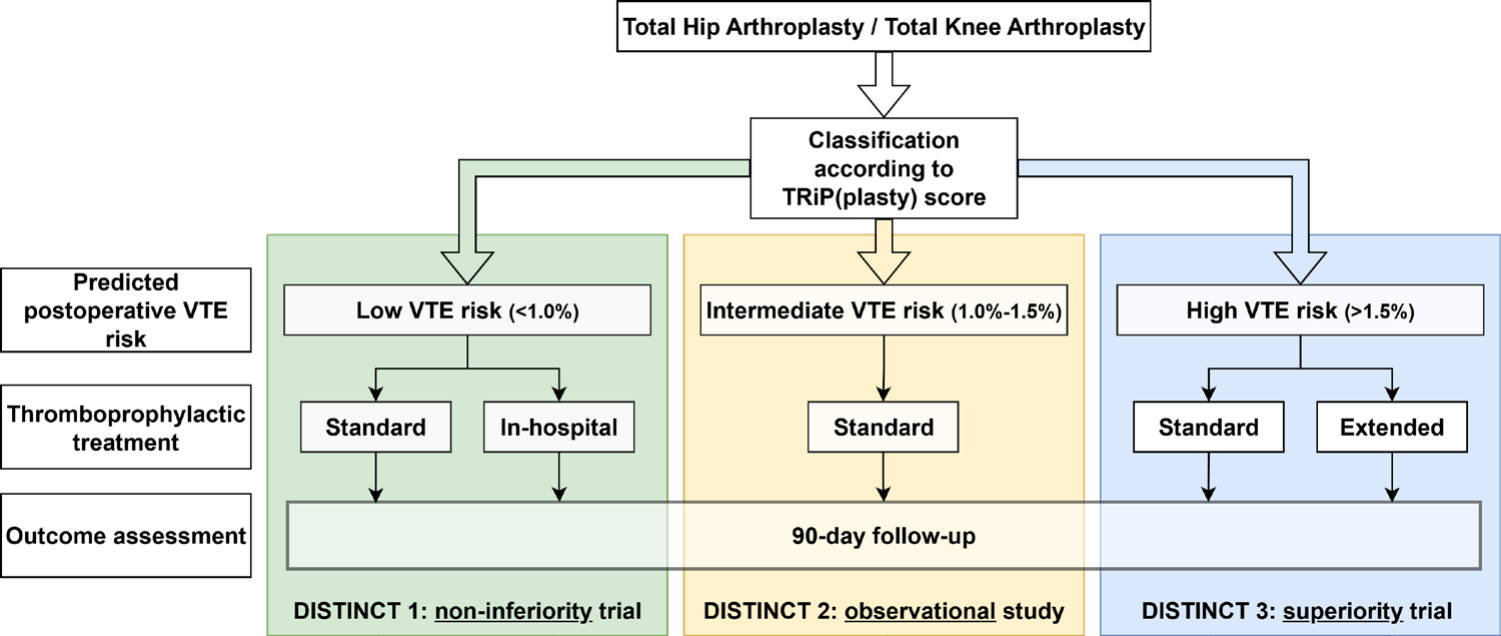
Study design: DISTINCT (inDividual, targeted thrombosIS prophylaxis versus the standard ‘one-size-fits-all’ approach in patients undergoing Total hIp or total kNee replaCemenT) trial. TRiP(plasty) score, Thrombosis Risk Prediction following total hip and knee arthroplasty score; VTE, venous thromboembolism.

**Figure 2:**
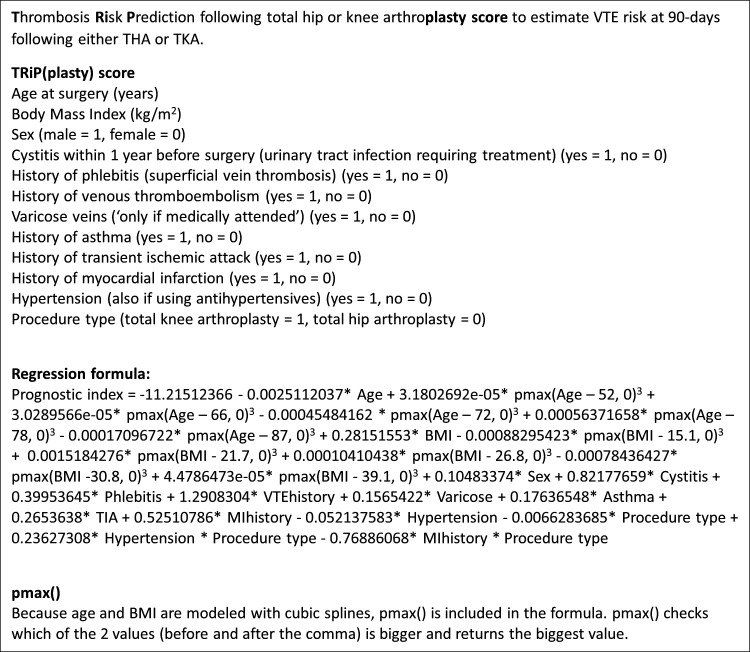
TRiP(plasty) model.^18^ The 90-day postoperative VTE risk can be calculated using the PI. The estimated individual postoperative VTE risk, as a percentage, can be calculated using the formula: exp(PI/(1+exp(PI))). BMI, body mass index; MI, myocardial infarction, PI, prognostic index; THA, total hip arthroplasty; TIA, transient ischaemic attack; TKA, total knee arthroplasty; TRiP(plasty) score, Thrombosis Risk Prediction following total hip and knee arthroplasty score; VTE, venous thromboembolism.

The study will consist of three study arms (figure 1) to which patients will be allocated based on their predicted VTE risk, as calculated by the TRiP(plasty) score (figure 2).^18^ Patients will either be assigned to the low VTE risk study arm: DISTINCT-1 trial (predicted 90-day postoperative VTE risk <1%), the intermediate VTE risk study arm: DISTINCT-2 study (predicted VTE risk ≥1% and ≤1.5%) or the high VTE risk study arm: DISTINCT-3 trial (predicted VTE risk >1.5%). Patients in the DISTINCT-1 trial will be randomised to either short-duration thromboprophylaxis (in-hospital only) or the control treatment (standard thromboprophylaxis). This study arm will be analysed as a non-inferiority trial. In the DISTINCT-2 study, participants will be treated with standard thromboprophylaxis. This study arm will be observational. Participants in the DISTINCT-3 trial will be randomised between the intervention (higher dose and longer duration thromboprophylaxis) and the control treatment (standard thromboprophylaxis). This study arm will be analysed as a superiority trial. All primary outcomes will be assessed up to 90 days following surgery. During the study, trial outcomes will be regularly evaluated by an independent Data and Safety Monitoring Board (DSMB). The Standard Protocol Items: Recommendations for Interventional Trials guidelines were followed when drafting the study protocol.^19 20^

### Eligibility criteria

Patients are eligible for the study if they undergo elective primary THA or TKA in one of the participating centres and meet the criteria listed below.

Inclusion criteria:

- Elective THA or TKA.
- Aged 18 years or older.

Exclusion criteria:

- Primary arthroplasty for fractures.
- Revision surgery.
- Hemiarthroplasty.
- Pregnancy.
- Current use of therapeutic anticoagulant therapy of any type (eg, Low-Molecular-Weight Heparin (LMWH), Direct Oral Anticoagulant (DOAC) or vitamin K antagonist).
- A contraindication for either study drug/treatment.
- Insufficient knowledge of the Dutch language.
- Insufficient mental or physical ability to fulfil trial requirements.
- Active malignancy (ie, cancer diagnosis or anti-cancer treatment within 6 months before surgery, excluding basal-cell or squamous-cell carcinoma of the skin).
- Patients using platelet aggregation inhibitors that cannot be temporarily discontinued at the discretion of their treating physician.

### Patient screening and recruitment

Every patient who is scheduled to undergo THA/TKA at one of the participating centres will be screened by a trained study staff member. In case of eligibility, an individual’s postoperative VTE risk is predicted by the TRiP(plasty) score. Accordingly, patients will be assigned to either the DISTINCT-1, DISTINCT-2 or DISTINCT-3 studies and will receive telephonic and digital or written study information (online supplemental files 1-3). Patients willing to participate will provide digital or written informed consent.

Based on the validation study of the TRiP(plasty) score, the expected distribution of patients over DISTINCT-1, DISTINCT-2 and DISTINCT-3 is 55%, 30% and 15%, respectively.^18^ We aim to include a minimum of 10 participating centres, which collectively perform approximately 17 150 THA/TKA procedures annually, ensuring the feasibility of the study.

### Randomisation, blinding and treatment allocation

Approximately 2 weeks before surgery, patients will be randomised centrally by a clinical data management system (Castor Electronic Data Capture (EDC), V.2025.2.4.0 Amsterdam, the Netherlands). Randomisation within the DISTINCT-1 and DISTINCT-3 trials will be performed in a 1:1 ratio, using block randomisation with variable block sizes and stratified by study centre. This is an open-label study, meaning that patients and treating physicians will not be blinded to the intervention; however, outcome assessors will be. The DISTINCT-2 study is an observational study arm.

### Study interventions

For the DISTINCT-1 trial, patients will be randomised between:

- Intervention
  In-hospital thromboprophylaxis only: any type of LMWH or DOAC in a prophylactic dose, as approved by the Dutch antithrombotic guidelines, administered until hospital discharge.^21^ The first dose of LMWH should be administered within 6–24 hours following surgery; the first dose of apixaban within 12–24 hours; the first dose of rivaroxaban within 6–10 hours and the first dose of dabigatran within 1–4 hours. The type of anticoagulant administered according to the local protocol (the same as that used in the control group). Patients undergoing outpatient THA or TKA (surgery and discharge on the same day) will receive one dose of thromboprophylaxis.
- Control
  Standard thromboprophylaxis for 4 weeks: any type of LMWH or DOAC in a prophylactic dose, as approved by the Dutch guidelines.^21^ The first dose of LMWH should be administered within 6–24 hours following surgery; the first dose of apixaban within 12–24 hours; the first dose of rivaroxaban within 6–10 hours and the first dose of dabigatran within 1–4 hours. The type of anticoagulant administered is according to the local protocol.

DISTINCT-2 study

- Standard thromboprophylaxis for 4 weeks: any type of LMWH or DOAC in a prophylactic dose, as approved by the Dutch guidelines.^21^ The first dose of LMWH should be administered within 6–24 hours following surgery; the first dose of apixaban within 12–24 hours; the first dose of rivaroxaban within 6–10 hours and the first dose of dabigatran within 1–4 hours. The type of anticoagulant administered is according to the local protocol.

For the DISTINCT-3 trial, patients will be randomised between:

- Intervention
  - Days 0–2: any type of LMWH or DOAC in a prophylactic dose, as approved by the Dutch guidelines.^21^ The first dose of LMWH should be administered within 6–24 hours following surgery; the first dose of apixaban within 12–24 hours; the first dose of rivaroxaban within 6–10 hours and the first dose of dabigatran within 1–4 hours following surgery. The type of anticoagulant administered is according to the local protocol.
  - Day 3: apixaban, 5 mg, two times per day, continued until 6 weeks after surgery. The start of the therapy is conditional on the fact that no active bleeding of the surgical wound can be observed. In the case of active bleeding, the prophylactic dose of thrombosis prophylaxis is continued. Thereafter, when no active bleeding has been observed for 24 hours following the bleeding, the 5 mg, two times per day, apixaban treatment will be initiated (off note, the total duration of thromboprophylaxis will not be extended and is maximised up to 6 weeks following surgery).

The timing of the first dose of apixaban is dependent on the type of preceding thromboprophylaxis during days 0–2: the first dose of study medication (apixaban) must be administered within 12–24 hours following the last dose of the initial provided thromboprophylaxis. The 5 mg, two times per day, apixaban dose will be adjusted to 2.5 mg, two times per day, in case of impaired kidney function (defined as estimated Glomerular Filtration Rate (eGFR) 15–30 mL/min). The use of any platelet aggregation inhibitors should be discontinued 5 days prior to surgery until 6 weeks postoperative.

- Control
  Standard thromboprophylaxis for 4 weeks: any type of LMWH or DOAC in a prophylactic dose, as approved by the Dutch guidelines.^21^ The first dose of LMWH should be administered within 6–24 hours following surgery; the first dose of apixaban within 12–24 hours; the first dose of rivaroxaban within 6–10 hours and the first dose of dabigatran within 1–4 hours. Thromboprophylaxis is continued until 4 weeks after surgery. The applied type of anticoagulant is according to the local protocol.

### Study outcomes

#### Primary outcomes

The primary efficacy outcome is the incidence of symptomatic VTE within 90 days following surgery. Symptomatic VTE is defined according to the International Society on Thrombosis and Haemostatis (ISTH) criteria.^22^ DVT is objectively confirmed by the presence of thrombotic material in the lumen of one or more deep veins of the leg. PE is objectively confirmed by the presence of thrombotic material in the lumen of a pulmonary vein.

The primary safety outcome is the incidence of major bleeding within 90 days following surgery. Bleeding is defined as major if it fulfils one of the ISTH criteria listed below, as described by Schulman *et al*^23^:

1. Fatal bleeding; and/or
2. Bleeding that is symptomatic and occurs in a critical area or organ, such as intracranial, intraspinal, intraocular, retroperitoneal, pericardial, a non‐operated joint or intramuscular with compartment syndrome, assessed in consultation with the surgeon; and/or
3. Extrasurgical site bleeding causing a fall in haemoglobin level of 20 g/L (1.24 mmol/L) or more, or leading to transfusion of two or more units of whole blood or red cells, with a temporal association within 24–48 hours of the bleeding and/or
4. Surgical site bleeding that requires a second intervention—open, arthroscopic or endovascular.
5. Surgical site bleeding that is unexpected and prolonged and/or sufficiently large to cause haemodynamic instability. There should be an associated fall in haemoglobin level of at least 20 g/L (1.24 mmol/L) or transfusion, indicated by the bleeding, of at least two units of whole blood or red cells, with temporal association within 24 hours of the bleeding.

#### Secondary outcomes

- CRNMB: any non-major bleeding requiring medical intervention by a healthcare professional, and/or leading to hospitalisation or an increased level of care and/or prompting a face-to-face evaluation.
- Prosthetic joint infection (PJI), defined according to the European Bone and Joint Society, as described by McNally *et al.*^24^
- Myocardial infarction, ischaemic stroke and death.
- Patient-reported outcome measures (EQ-5D (EuroQol 5-Dimension Quality of Life Questionnaire), Oxford Hip Score,^25^ Oxford Knee Score,^26^ Visual Analogue Scale (VAS) pain).
- Impact of events on quality-adjusted life years (QALYs) and societal costs.

### Study follow-up

Primary and secondary outcomes will be assessed using a questionnaire at 2 weeks, 6 weeks and 90 days postoperatively. Additionally, patients who experience a symptomatic VTE, major bleed or PJI receive an additional questionnaire at 1 year postoperatively. Controls, 1:1 matched on age, sex, study centre and type of surgery, will receive the same questionnaire. All postoperative care is according to the current standard of care; no routine VTE screening will be performed. In the case of a (possible) outcome event, additional data on the event, including imaging studies, consult letters, discharge letters and any other relevant information, are collected from the medical record of the participant. In table 1, study procedures and assessments are presented.

**Table 1:** An overview of study procedures and assessments

Evaluation	Preoperative	Day 0 (surgery)	2 weeks	6 weeks	90 days	12 months
Baseline characteristics, including age, sex, BMI, comorbidities and VTE risk factors	X					
Perioperative outcomes*		X				
Outcome event(s) by questionnaire			X	X	X	
Treatment compliance (questionnaire)				X		
VAS	X				X	
OHS/OKS	X					
EQ-5D	X				X	
In case of an outcome event(s) and controls						
OHS/OKS						X
SF-36						X
EQ-5D						X

* Perioperative variables are derived from the Dutch Arthroplasty Registry (LROI).

BMI, body mass index; EQ-5D, EuroQol 5-Dimensions Quality of Life Questionnaire; LROI, Landelijke Registratie Orthopedische Interventies; OHS, Oxford Hip Score; OKS, Oxford Knee Score; SF-36, 36-Item Short Form Survey; VAS, Visual Analogue Scale; VTE, venous thromboembolism.

### Sample size

#### Sample size calculation: DISTINCT-1 trial

By only including patients with a predicted VTE risk of <1.0%, a 3-month cumulative incidence of symptomatic VTE of 0.75% is expected in the control arm. This risk is based on the results from the development and validation studies of the TRiP(plasty) score.^18^ Multiple observational studies among patients in fast-track treatment protocols have shown that the risk of postoperative symptomatic VTE is ≤1% or even <0.5% with the use of in-hospital thromboprophylaxis only.^6 8 10 11^ Therefore, we expect no reduced or increased VTE risk when thromboprophylaxis is limited to in-hospital use only. Hence, the expected risk in the short-duration prophylaxis group is also 0.75%. The non-inferiority limit is set at 1%, which represents the minimal clinically important difference.^27^ Considering a one-sided alpha of 0.025 and a power of 90%, this leads to a necessary sample size of 3130 patients. To account for a maximum dropout rate of 10%, we aim to include 1739 patients in each group, totalling 3478 patients.

The incidence of (any) bleeding following THA/TKA has been reported to range from 0.5% to 3%. This variation is mainly caused by differences in bleeding definitions and the completeness and accuracy of patient follow-up.^12 13^ Hence, exact estimations of bleeding risks are difficult to obtain. Based on the available literature, we estimate that shortening the duration of thromboprophylaxis has no effect on major bleeding rates. The estimated relative reduced risk for minor bleeding+CRNMB is approximately 29%–50%.^10^,^1228 29^

#### Sample size calculation: DISTINCT-2 study

In the intermediate VTE risk group, the cumulative incidence of symptomatic VTE within 90 days is expected to be 1.3%. With n=2500, the width of the 95% CI is expected to be 0.9% to 1.7%, with a probability of less than 15% that the upper bound of a two-sided 95% CI will exceed the 2% margin. We consider this a sufficiently precise estimate of VTE risk in this patient group.

#### Sample size calculation: DISTINCT-3 trial

By only including patients with a predicted VTE risk of >1.5%, a 90-day cumulative incidence of symptomatic VTE of 2.5% is expected in the control arm. This risk is based on the results from the development and validation studies of the TRiP(plasty) score.^18^ We expect that the intervention gives a relative risk reduction of 50% (ie, expected outcome in the intervention arm: 1.25%). This risk reduction was estimated from earlier dose-finding trials and our recently performed pilot study.^30^,^32^

At a two-sided alpha level of 0.05 and a power of 80%, 3694 patients would be necessary. To account for the interim analysis and thus testing against a slightly stricter statistical significance level at the final analysis, a total of 3748 patients are required (1874 per treatment arm). To account for a maximum dropout rate of approximately 9%, we aim to include 2050 patients in each arm, totalling 4100 patients.

For the DISTINCT-3 trial, we assume an increased relative major bleeding risk of 65% (from 0.5% to 0.83%) by increasing the thromboprophylaxis dose and duration. This estimate is based on results from earlier trials and our pilot study.^30^,^32^

### Statistical analysis

Both modified intention-to-treat (ITT) and per-protocol (PP) analyses will be performed.

#### Intercurrent events

Randomised patients who did not undergo surgery will be excluded from the ITT analysis. Treatment discontinuation will be ignored in the primary analysis. In a secondary PP analysis, we will only include patients who completely adhered to the study protocol. This means that they are not allowed to skip study medication. If this happens, patients will be excluded from the secondary analysis. We will report the proportion of patients who did not adhere to the study protocol, including details on the number of skipped dosages and the reasons for non-adherence.

Death as an intercurrent event will be included as an outcome event in the analyses (both in ITT and PP) if the outcome adjudication committee adjudicates this death as an outcome event (death related to a fatal PE or bleed). If death is not related to VTE or bleeding, it will be ignored in the analyses (as such an event is very rare within 90 days following surgery).

### Analysis of primary endpoints

#### DISTINCT-1 statistics

A primary modified ITT analysis and a secondary PP analysis will be performed. The overall cumulative incidences of symptomatic VTE and major bleeding between the control and intervention groups will be compared, using a one-sided non-inferiority analysis for the absolute risk difference, with a significance level set as a one-sided p value of 0.025.

#### DISTINCT-2 statistics

Overall cumulative incidences for symptomatic VTE and major bleeding for the whole cohort will be estimated with a 95% CI.

#### DISTINCT-3 statistics

A primary modified ITT analysis and a secondary PP analysis will be performed. The overall cumulative incidences of symptomatic VTE and major bleeding between the control and the intervention groups will be compared, using a superiority analysis for the risk difference. The 95% CI for the absolute difference in event risks will be determined.

### Analysis of secondary endpoints

Risks of CRNMB, minor bleeding, PJI, myocardial infarction, ischaemic stroke and death will be estimated and compared between groups in a similar fashion. Functional and quality-of-life outcomes at 1 year postoperatively in patients who experienced symptomatic VTE, major bleeding or PJI and in controls will be compared between groups, across all groups.

We expect that the intervention will improve health outcomes at lower healthcare and societal costs. A cost-utility analysis will be performed using a Markov model to estimate the lifelong impact of VTE and bleeding events on discounted QALYs and societal costs. The intervention group will be compared with the control group according to ITT, separately for the DISTINCT-1 trial and the DISTINCT-3 trial.

The 3-month symptomatic VTE and bleeding rates (from the primary analysis) will be estimated from the trial. The impact per event on QALYs (ie, estimated using the EQ-5D), as well as on healthcare and productivity costs, will be estimated from the differences in patient-reported questionnaires preoperatively and after 14 days, 30 days, 90 days and 12 months. Medication and other healthcare will be valued using Dutch reference prices. QALYs and costs will be compared using net-benefit analysis, taking data and parameter uncertainty into account and with multiple imputation to account for missing data. Sensitivity and scenario analyses will include perspective (societal vs healthcare) and the valuation of productivity (friction costs vs human capital approach).^33^

### Interim analyses and patient safety

A DSMB will be installed prior to the start of the study. The DSMB will meet and review the progress and acquired data. Interim analyses for the DISTINCT-1 trial and DISTINCT-3 trial are planned after 25%, 50% and 75% of the target number of subjects have been recruited and completed a 90-day follow-up. The DISTINCT-1 trial will be terminated when the lower limit of a two-sided 95% CI for the VTE incidence estimates in either of the two study groups exceeds 0.75%. In the DISTINCT-3 trial, we will test superiority using the O’Brien-Fleming alpha spending function to control the type I error; that is, we will perform a two-sided test, where superiority will be concluded in case the absolute value of the test statistic exceeds the boundary level of 4.084, 2.888 and 2.358 for the 25%, 50% and 75% interim analyses, respectively. In the superiority analysis, at the end of the study (after inclusion of the planned number of participants), superiority will be concluded if the absolute value of the test statistic exceeds the boundary level of 1.98. In addition, as a main safety rule, the absolute risk for major bleeds must not exceed the absolute risk for symptomatic VTE in either of the two treatment arms. The DSMB reviews the interim analyses and provides advice on the conduct of the trial to the trial steering committee.

### Patient and public involvement

A patient representative is part of the trial steering committee. As such, patients are involved in all aspect this study.

## Ethics and dissemination

This study will be conducted in accordance with the Declaration of Helsinki and applicable guidelines, laws and acts. The study is registered at ClinicalTrials.gov (NCT06581965) prior to the start of participant inclusion.

Ethical approval is provided by the Medical Research Ethics Committee Leiden-Den Haag-Delft (MREC LDD; EU trial number 2023-510186-98). MREC LDD will be notified in case of any substantial amendments; additionally, in case of an important protocol modification, the trial registry will be updated. Trial results will be published in international peer-reviewed journals regardless of the study findings.

Data will be collected in accordance with the General Data Protection Regulations. After data collection and data cleaning are completed, de-identified data will be registered in a repository and made available for further research on reasonable request to the corresponding author.

## Supplementary material

10.1136/bmjopen-2025-101180 online supplemental file 1

10.1136/bmjopen-2025-101180 online supplemental file 2

10.1136/bmjopen-2025-101180 online supplemental file 3
