# Supplementary material for: Study protocol for the DISTINCT trial: inDividual, targeted thrombosIS prophylaxis versus the standard ‘one-size-fits-all’ approach in patients undergoing Total hIp or total kNee replaCemenT – a national, multicentre, randomised, multiarm, open-label trial
Source: BMJ Open. 2025 Oct 6;15(10):e101180. doi: 10.1136/bmjopen-2025-101180 (PMC12506130; doi:10.1136/bmjopen-2025-101180)
Supplement: online supplemental file 2 [file bmjopen-15-10-s002.pdf]

Leids Universitair Medisch Centrum

Proefpersoneninformatie voor deelname aan medisch-wetenschappelijk onderzoek

Verbeteren van de behandeling om trombose te voorkomen na een heup of knie vervangende operatie

Officiële titel: Geïndividualiseerde tromboseprofylaxe voor patiënten die een heup of knie vervangende operatie ondergaan: een nationale, multicenter, gerandomiseerde, multi-arm, open label trial.

Een groot Nederlands onderzoek in meerdere ziekenhuizen, waarin de mogelijke voor- en nadelen worden onderzocht van een geïndividualiseerde dosering bloedverdunners bij mensen na een totale heup of knie vervangende operatie.

Inleiding

Geachte heer/mevrouw,

Met deze informatiebrief willen we u vragen of u wilt meedoen aan medisch-wetenschappelijk onderzoek. Meedoen is vrijwillig. U krijgt deze brief omdat u binnenkort een heup of knie vervangende operatie ondergaat.
U leest hier om wat voor onderzoek het gaat, wat het voor u betekent, en wat de voordelen en nadelen zijn. Het is veel informatie. Wilt u de informatie doorlezen en beslissen of u wilt meedoen? [Als u wilt meedoen, kunt u het formulier onderaan de pagina digitaal invullen en ondertekenen.] [Als u wilt meedoen, kunt u het formulier invullen dat u vindt in bijlage C.]

Stel uw vragen

U kunt uw beslissing nemen met de informatie die u in deze informatiebrief vindt. Daarnaast raden we u aan om dit te doen:
- Stel vragen aan de onderzoeker die u deze informatie geeft.
- Praat met uw partner, familie of vrienden over dit onderzoek.
- Lees de informatie op www.rijksoverheid.nl/mensenonderzoek.

1. Algemene informatie

Het Leids Universitair Medisch Centrum (LUMC) heeft dit onderzoek opgezet. Hieronder noemen we het LUMC steeds de ‘opdrachtgever’. Onderzoekers, dit kunnen artsen, onderzoekers en onderzoeksverpleegkundigen zijn, voeren het onderzoek uit in verschillende ziekenhuizen. Het [naam deelnemend ziekenhuis] is één van de ziekenhuizen dat deelneemt aan dit onderzoek.

Deelnemers aan een medisch-wetenschappelijk onderzoek worden vaak proefpersonen genoemd. Zowel patiënten als mensen die gezond zijn, kunnen proefpersoon zijn.

Voor dit onderzoek zijn ongeveer 10.000 proefpersonen nodig, verspreid over meerdere ziekenhuizen in Nederland.

De medisch-ethische toetsingscommissie Leiden Den Haag Delft heeft dit onderzoek goedgekeurd.

2. Wat is het doel van het onderzoek?

Het doel van de studie is het verbeteren van de behandeling met bloedverdunners om te zorgen dat er geen trombosebeen of longembolie ontstaat (samen ook wel veneuze trombose genoemd) na een heup of knie vervangende operatie. In de studie zal de behandeling worden aangepast op basis van uw medische gegevens om de balans tussen de kans op veneuze trombose en de kans op een bloeding te verbeteren. Te weinig bloedverdunners vergroot namelijk de kans op trombose en teveel bloedverdunners verhogen de kans op een bloeding. Behandeling met een juiste hoeveelheid bloedverdunners is dus belangrijk om zowel trombose als bloedingen te voorkomen.

In deze studie, met de naam DISTINCT 2, krijgt u na de operatie de standaardbehandeling volgens de huidige Nederlandse richtlijn, namelijk een lage dosering bloedverdunners tijdens de ziekenhuisopname en thuis tot 4 weken na de operatie. Wij zullen u na de operatie volgen.

3. Video met studie informatie

[Als u op de onderstaande link klikt komt u bij een video op de website van het LUMC waarin kort wordt verteld wat er in deze brief staat. Lees ook de rest van de brief goed door. De informatie in de video is geen vervanging van de informatie in deze brief. Klik op de onderstaande link om naar de website van het LUMC te gaan waar de video te zien is.] (versie digitaal)

[Typ de link over of scan de QR-code hieronder om naar de website van het LUMC te gaan. Hier staat een video waarin kort wordt verteld wat er in deze brief staat. Lees ook de rest van de brief goed door. De informatie in de video is geen vervanging van de informatie in deze brief.] (versie papier)

4. Wat is de achtergrond van het onderzoek?

Een knie of heup vervangende operatie geeft een verhoogde kans op het ontwikkelen van veneuze trombose. Veneuze trombose is een ziekte waarbij er een bloedstolsel vast komt te zitten in een bloedvat. Dit kan optreden in de benen of in de longen. Dat wordt een

trombosebeen of longembolie genoemd. Veneuze trombose is de verzamelnaam voor een trombosebeen en longembolie.

Om te zorgen dat mensen die een knie of heup vervangende operatie ondergaan geen veneuze trombose krijgen, geven artsen bloedverdunners na de operatie. Dit kan zijn in de vorm van tabletten of een dagelijkse injectie onder de huid. Helaas krijgt ondanks deze bloedverdunners nog steeds een deel van de mensen een veneuze trombose. Daarnaast krijgt ook een deel van de mensen na de operatie een bloeding.

Op dit moment krijgen alle mensen die een knie of heup vervangende operatie ondergaan dezelfde hoeveelheid bloedverdunner. Maar niet alle mensen zijn hetzelfde. Dit betekent dat voor een deel van de mensen de bloedverdunners niet nodig zijn, omdat zij ook zonder deze medicijnen geen veneuze trombose zouden krijgen. Bij deze mensen doen de bloedverdunners waarschijnlijk meer schade (in de vorm van bloedingen) dan dat zij goed doen. Aan de andere kant zijn er mensen die ondanks de bloedverdunners toch veneuze trombose krijgen. Bij deze mensen zijn de bloedverdunners niet voldoende om te zorgen dat ze geen veneuze trombose krijgen en dit betekent dat er iets extra’s nodig is.

Tot voorheen was het niet goed mogelijk om verschil te maken tussen deze groepen mensen. Hierdoor worden op dit moment alle patiënten op dezelfde manier met bloedverdunners behandeld.

Sinds enige tijd kan er aan de hand van medische gegevens een nauwkeurige voorspelling gemaakt worden van de kans dat deze mensen na de operatie een veneuze trombose krijgen. Deze voorspelling wordt berekend met behulp van een voorspellingsmodel. Aan de hand van deze voorspelling kan de behandeling met bloedverdunners aangepast worden. Dit om de balans tussen de kans op het krijgen van veneuze trombose en de kans op bloedingen als gevolg van de bloedverdunning te verbeteren.

5. Hoe verloopt het onderzoek?

Hoelang duurt het onderzoek?

Doet u mee met het onderzoek? De duur van het onderzoek is voor de meeste mensen 3 maanden na uw operatie. Als u een trombose, bloeding of infectie krijgt, ontvangt u 12 maanden na de operatie nog 1 keer een vragenlijst.

Stap 1: bent u geschikt om mee te doen?

We willen eerst weten of u geschikt bent om mee te doen. Daarom zullen uw behandelend specialist en de onderzoeker beoordelen of er in uw medische geschiedenis redenen zijn waarom u niet mee zou kunnen doen. Dit is bijvoorbeeld het geval wanneer u al een hoge dosering bloedverdunners gebruikt vanwege een aandoening zoals een hartritmestoornis.

Stap 2: de heup of knie vervangende operatie
Als u deelneemt aan de studie blijft uw operatie hetzelfde. Ook de zorg na uw operatie verandert niet.

Stap 3: de behandeling met bloedverdunners
Alle deelnemers van de DISTINCT 2 studie krijgen de standaard behandeling volgens de huidige Nederlandse richtlijn, namelijk een lage dosering bloedverdunners tijdens de ziekenhuisopname en thuis tot 4 weken na de operatie. Via een vragenlijst doen we onderzoek naar de risicofactoren voor veneuze trombose en bloedingen.

Stap 4: onderzoeken en metingen
Bij deelname aan dit onderzoek wordt u vanaf de operatie 90 dagen (3 maanden) gevolgd. Als u een trombose, bloeding of infectie krijgt wordt u 1 jaar opgevolgd. Gedurende deze tijd zijn er *geen extra controles of metingen nodig naast de gebruikelijke afspraken* die passen bij de nazorg van de operatie.

U krijgt wel in totaal vier keer een vragenlijst toegestuurd per email of post. Deze vragenlijsten zullen vanuit het LUMC worden verzonden. In deze vragenlijsten vragen we naar uw ervaringen met het gebruik van de bloedverdunner. Daarnaast vragen we ook naar mogelijke bijwerkingen en of u toch een veneuze trombose of een bloeding heeft gehad. Ook vragen we met uw toestemming de gegevens van uw huisarts of uit het ziekenhuis op. Deelnemers die een trombose, bloeding of infectie hebben gehad krijgen een extra vragenlijst 1 jaar na de operatie. Ook een aantal deelnemers die geen trombose, bloeding of infectie hebben gehad, zullen wij vragen om na 1 jaar een eenmalige vragenlijst in te vullen. U kunt hiervoor benaderd worden.

Rondom de operatie wordt u door uw ziekenhuis gevraagd om vragenlijsten in te vullen over uw knie of heup. Deze vragenlijsten horen niet bij het onderzoek, maar worden verstuurd naar iedereen die een knie of heup vervangende operatie ondergaat. De antwoorden van deze vragenlijsten zullen ook gebruikt worden voor dit onderzoek.

In het onderstaande stroomschema ziet u het verloop van de studie voor u.

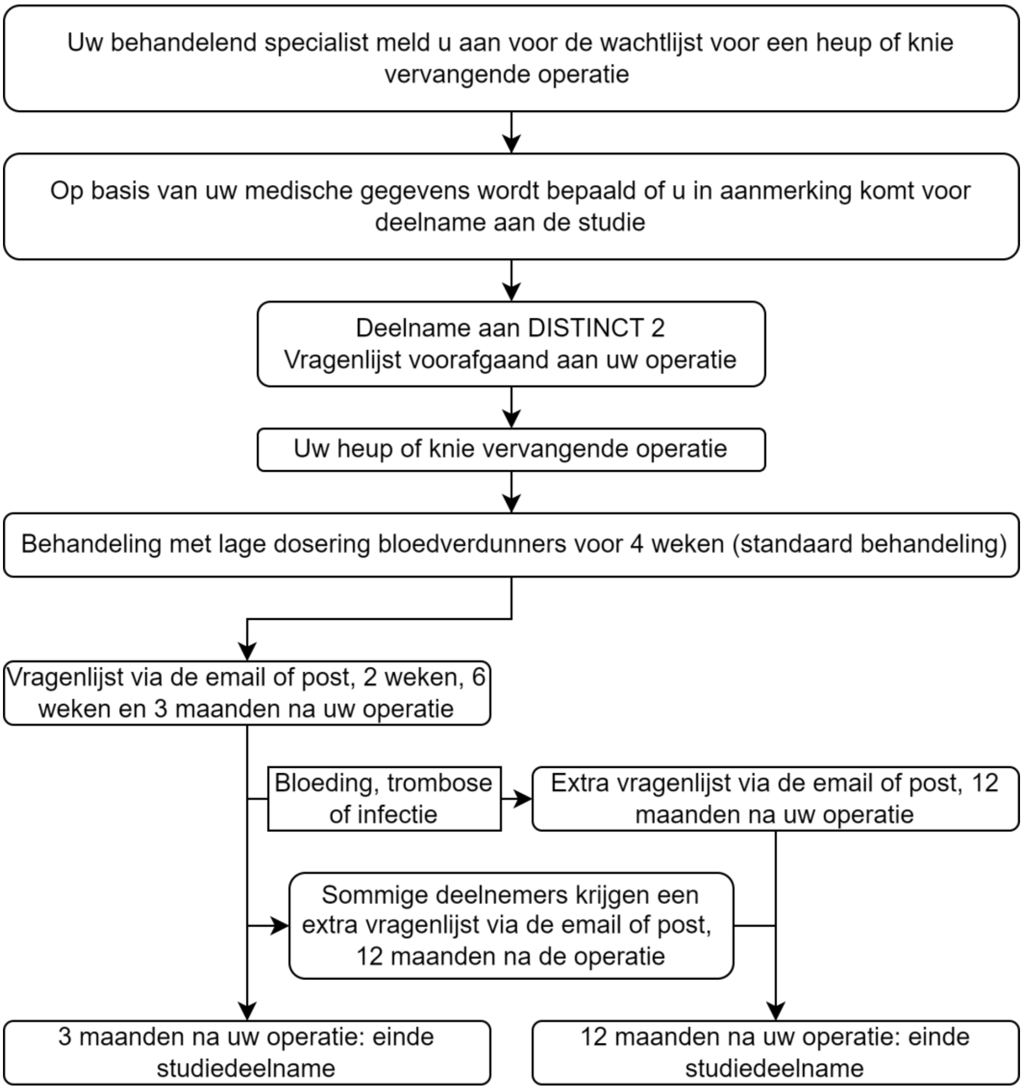

6. Welke afspraken maken we met u?

We willen graag dat het onderzoek goed verloopt. Daarom maken we de volgende afspraken met u:

- U neemt de bloedverdunner op de manier die de arts/verpleegkundige of onderzoeker u heeft uitgelegd.
- U doet tijdens dit onderzoek niet ook nog mee aan een ander medisch-wetenschappelijk onderzoek over veneuze trombose, bloedingen of geneesmiddelen. In overleg met de onderzoekers kan hier een uitzondering op gemaakt worden.
- U neemt contact op met de onderzoeker in deze situaties:

- U krijgt plotseling problemen met uw gezondheid.
- U wilt niet meer meedoen met het onderzoek.

Mag u of uw partner zwanger worden tijdens het onderzoek?
Vrouwen die zwanger zijn of borstvoeding geven, kunnen niet meedoen aan dit onderzoek. Vrouwen mogen ook niet zwanger worden tijdens het onderzoek. Dit onderzoek kan namelijk gevolgen hebben voor een ongeboren kind.

7. Van welke bijwerkingen, nadelige effecten of ongemakken kunt u last krijgen?

De volgende bijwerkingen komen vaak voor bij gebruik van bloedverdunners (1 op de 100 tot 9 op de 100 patiënten):

- Bloedingen (dit kunnen spontane bloedingen zijn, maar ook de kans op niet spontane bloedingen na bijvoorbeeld een val is verhoogd)
- Bloedarmoede

Andere mogelijke bijwerkingen staan in de bijsluiter van de specifieke bloedverdunner die u voorgeschreven krijgt. Deze ontvangt u van de apotheek bij het afhalen van de bloedverdunner.

8. Wat zijn de voordelen en de nadelen als u meedoet aan het onderzoek?

Meedoen aan het onderzoek kan voordelen en nadelen hebben. Hieronder zetten we ze op een rij. Denk hier goed over na, en praat erover met anderen.

De behandeling met bloedverdunners in de studie is gelijk aan de gebruikelijke behandeling met bloedverdunners volgens de Nederlandse richtlijn, de deelname heeft voor u geen voordelen of nadelen.

Wilt u niet meedoen?
U beslist zelf of u meedoet aan het onderzoek. Wilt u niet meedoen? Dan krijgt u de gewone behandeling voor het voorkomen van veneuze trombose na een knie of heup vervangende operatie. Dit betekent dat u een lage dosis bloedverdunners krijgt voor de duur die gebruikelijk is in uw ziekenhuis.. Uw arts kan u hier meer over vertellen. En over de voor- en nadelen daarvan.

9. Wanneer stopt het onderzoek?

De onderzoeker laat het u weten als er nieuwe informatie over het onderzoek komt die belangrijk voor u is. De onderzoeker vraagt u daarna of u blijft meedoen.

In deze situaties stopt voor u het onderzoek:

- Het einde van het hele onderzoek is bereikt. Dit is 90 dagen na de operatie. Alleen als u een trombose, bloeding of infectie krijgt of u gevraagd wordt voor de controle groep krijgt u na 1 jaar één extra vragenlijst.
- U bent zwanger geworden.
- U wilt zelf stoppen met het onderzoek. Dat mag op ieder moment. Meld dit dan meteen bij de onderzoeker. U hoeft er niet bij te vertellen waarom u stopt. U krijgt dan weer de gewone behandeling voor het voorkomen van veneuze trombose na een knie of heup vervangende operatie.
- De onderzoeker vindt het beter voor u om te stoppen.
- Een van de volgende instanties besluit dat het onderzoek moet stoppen:
  - het LUMC
  - de overheid, of
  - de medisch-ethische commissie die het onderzoek beoordeelt.

Wat gebeurt er als u stopt met het onderzoek?
De onderzoekers gebruiken de gegevens die tot het moment van stoppen zijn verzameld.

Het hele onderzoek is afgelopen als alle deelnemers klaar zijn.

10. Wat gebeurt er na het onderzoek?

Kunt u de medicijnen blijven gebruiken?
Na het onderzoek is er geen reden meer om de bloedverdunners te gebruiken ter voorkoming van een veneuze trombose als gevolg van de operatie. Indien u om een andere medische reden bloedverdunners gebruikt, zal uw arts u hierover informeren.

Krijgt u de resultaten van het onderzoek?
Na uw deelname laat de onderzoeker u weten wat de belangrijkste uitkomsten zijn van het onderzoek. Het onderzoek zal in totaal ongeveer 6 jaar in beslag nemen. Het kan dus zijn dat u 6 jaar na u deelname pas de uitkomsten ontvangt.

11. Wat doen we met uw gegevens?

Doet u mee met het onderzoek? Dan geeft u ook toestemming om uw gegevens te verzamelen, gebruiken en bewaren.

Welke gegevens bewaren we?

We bewaren deze gegevens:

- uw naam
- uw geslacht
- uw adres of emailadres en telefoonnummer
- uw geboortedatum
- gegevens over uw gezondheid
- (medische) gegevens die we tijdens het onderzoek verzamelen

Waarom verzamelen, gebruiken en bewaren we uw gegevens?

We verzamelen, gebruiken en bewaren uw gegevens om de vragen van dit onderzoek te kunnen beantwoorden, om contact met u op te kunnen nemen en om de resultaten te kunnen publiceren.

Hoe beschermen we uw privacy?

Om uw privacy te beschermen geven wij uw gegevens een code. Op al uw gegevens zetten we alleen deze code. De sleutel van de code bewaren we op een beveiligde plek in het coördinerende centrum (het LUMC). Als we uw gegevens verwerken, gebruiken we steeds alleen die code. Ook in rapporten en publicaties over het onderzoek kan niemand terughalen dat het over u ging.

Uw toestemming

Het is voor dit onderzoek nodig dat de onderzoeker in het LUMC inzage heeft in uw persoonsgegevens. U geeft hiervoor apart toestemming op het toestemmingsformulier. In rapporten en publicaties over het onderzoek zijn de gegevens niet tot u te herleiden.

Wie kunnen uw gegevens zien?

Sommige personen kunnen wel uw naam en andere persoonlijke gegevens zonder code inzien. Dit zijn mensen die controleren of de onderzoekers het onderzoek goed en betrouwbaar uitvoeren. Deze personen kunnen bij uw gegevens komen:

- De onderzoekers uit het LUMC die de vragenlijsten versturen en zo nodig extra gegevens opvragen bij uw medisch specialist of huisarts in het geval u een trombose of bloeding krijgt.
- Leden van de commissie die de veiligheid van het onderzoek in de gaten houdt.
- Een controleur die door de onderzoeker is ingehuurd.
- Nationale en internationale toezichthoudende autoriteiten. Bijvoorbeeld de Inspectie Gezondheidszorg en Jeugd.

Deze personen houden uw gegevens geheim. Wij vragen u voor deze inzage toestemming te geven.

Hoelang bewaren we uw gegevens?
We bewaren uw gegevens 25 jaar na het einde van de studie in het LUMC. Deze termijn is verplicht voor onderzoek met geneesmiddelen.

Mogen we uw gegevens gebruiken voor ander onderzoek?
Wij willen uw gegevens graag gebruiken voor ander onderzoek. Op het toestemmingsformulier kunt u aangeven of u hiermee akkoord gaat. Onderzoeksgegevens zullen maximaal 25 jaar na het einde van het onderzoek bewaard blijven. U kunt deze toestemming altijd weer intrekken.

Kunt u uw toestemming voor het gebruik van uw gegevens weer intrekken?
U kunt uw toestemming voor het gebruik van uw gegevens op ieder moment intrekken. Maar let op: trekt u uw toestemming in, en hebben onderzoekers dan al gegevens verzameld voor een onderzoek? Dan mogen zij deze gegevens nog wel gebruiken.

Wilt u meer weten over uw privacy?

- Wilt u meer weten over uw rechten bij de verwerking van persoonsgegevens? Kijk dan op www.autoriteitpersoonsgegevens.nl.
- Heeft u vragen over uw rechten? Of heeft u een klacht over de verwerking van uw persoonsgegevens? Neem dan contact op met degene die verantwoordelijk is voor de verwerking van uw persoonsgegevens. Voor uw onderzoek is dat:
    - Het Leids Universitair Medisch Centrum. Zie bijlage A voor contactgegevens, en website.
- Als u klachten heeft over de verwerking van uw persoonsgegevens, raden we u aan om deze eerst te bespreken met het onderzoeksteam. Bekijk voor meer informatie over privacy het privacy statement van het LUMC op de LUMC-website: zie bijlage A U kunt ook naar de Functionaris Gegevensbescherming van het LUMC gaan. Of u dient een klacht in bij de Autoriteit Persoonsgegevens.

Landelijke registratie orthopedische implantaten (LROI)
Wij vragen uw toestemming voor het gebruik van gegevens uit de Landelijke registratie orthopedische implantaten (LROI). In de LROI wordt informatie over uw operatie geregistreerd ook worden hier de vragenlijsten opgeslagen die u rondom uw operatie krijgt van uw eigen ziekenhuis. Deze gegevens worden voor iedereen verzameld, ook voor mensen die niet meedoen aan het onderzoek. Voor meer informatie over de LROI kijk op: https://www.zorgvoorbeweging.nl/wetenschap/registratie-van-prothesen/

Waar vindt u meer informatie over het onderzoek?
Op de volgende websites vindt u meer informatie over het onderzoek: https://www.lumc.nl/distinct en https://clinicaltrials.gov/study/NCT06581965. Na het onderzoek kan de website een samenvatting van de resultaten van dit onderzoek tonen.

12. U krijgt geen vergoeding als u meedoet aan het onderzoek

De lage dosering met bloedverdunners krijgt u vergoed van uw zorgverzekeraar. Er zijn voor u geen kosten verbonden aan studiedeelname. Ook hoeft u niet extra in het ziekenhuis te komen voor studieafspraken. U krijgt daarom geen vergoeding als u meedoet aan dit onderzoek.

13. Bent u verzekerd tijdens het onderzoek?

Voor iedereen die meedoet aan dit onderzoek is een verzekering afgesloten. De verzekering betaalt voor schade door het onderzoek. Maar niet voor alle schade. In **bijlage B** vindt u meer informatie over de verzekering en de uitzonderingen. Daar staat ook aan wie u schade kunt melden.

14. We informeren uw huisarts en behandelend specialist

De onderzoeker stuurt uw behandelend specialist een bericht om te laten weten dat u meedoet aan het onderzoek.

15. Heeft u vragen?

Vragen over het onderzoek kunt u stellen aan het onderzoeksteam.
Heeft u een klacht? Bespreek dit dan met de onderzoeker of de arts die u behandelt. Wilt u dit liever niet? Ga dan naar de klachtenfunctionaris van het Leids Universitair Medisch Centrum. In bijlage A staat waar u die kunt vinden.

16. Hoe geeft u toestemming voor het onderzoek?

U kunt eerst rustig nadenken over dit onderzoek. Daarna vertelt u de onderzoeker of u de informatie begrijpt en of u wel of niet wilt meedoen. Wilt u meedoen? Dan vult u het toestemmingsformulier in dat u bij deze informatiebrief vindt. U en de onderzoeker krijgen allebei een getekende versie van deze toestemmingsverklaring.

Dank voor uw tijd.

17. Bijlagen bij deze informatie

A. Contactgegevens *<per deelnemend centrum aan te passen>*

B. Informatie over de verzekering

C Toestemmingsformulier

Bijlage A: contactgegevens voor [naam deelnemend centrum]

Contactgegevens [naam deelnemend centrum]
Lokaal onderzoekers:
[Lokaal hoofdonderzoeker met contactgegevens]

Contactpersonen:
[Lokaal contactpersoon met contactgegevens]

Contactgegevens betrokken onderzoekers LUMC
Onderzoeker: Dr. B. Nemeth. Verbonden aan de afdeling Klinische Epidemiologie & afdeling Orthopedische Chirurgie aan het Leids Universitair Medisch Centrum. Albinusdreef 2, 2300 RC Leiden.
Telefoonnummer: 071-5264037

Onderzoeksarts: Drs. R.Y. Kok. Verbonden aan de afdeling Klinische Epidemiologie aan het Leids Universitair Medisch Centrum. Albinusdreef 2, 2300 RC Leiden.
Telefoonnummer: 071-5263584

De onderzoekers en onderzoeksarts zijn ook te bereiken via het emailadres van de DISTINCT studie: DISTINCT@lumc.nl

Klachten:
[dienst of persoon met contactgegevens en bereikbaarheid, deelnemend centrum]

Functionaris voor de Gegevensbescherming van de instelling:
Bij vragen of klachten over de verwerking van uw persoonsgegevens raden we u aan eerst contact op te nemen met de onderzoek locatie. U kunt ook contact opnemen met de Functionaris voor de Gegevensbescherming van de instelling.

Functionaris gegevensbescherming:
[FG deelnemend centrum met contact gegevens]

Voor meer informatie over uw rechten:
Contactgegevens LUMC
Albinusdreef 2
2333 ZA Leiden
Centraal telefoonnummer: (071) 526 91 11
Voor meer informatie over uw rechten zie de website van het LUMC
https://www.lumc.nl/12367/Deelnemers-wetenschappelijk-onderzoek/

Bijlage B: informatie over de verzekering

Het Leids Universitair Medisch Centrum heeft een verzekering afgesloten voor iedereen die meedoet aan het onderzoek. De verzekering betaalt de schade die u heeft doordat u aan het onderzoek meedeed. Het gaat om schade die u krijgt tijdens het onderzoek, of binnen 4 jaar na het onderzoek. U moet schade binnen 4 jaar melden bij de verzekeraar.

Heeft u schade door het onderzoek? Meld dit dan bij deze verzekeraar:
U kunt contact opnemen met de verzekeraar Centramed zoals hieronder beschreven.

De verzekeraar van het onderzoek is:	
Naam:	Centramed
Adres:	Maria Montessorilaan 9, 2719 DB Zoetermeer
Telefoonnummer:	070-3017070
E-mail:	info@centramed.nl
Polisnummer:	624.530.305

De verzekering betaalt maximaal € *650.000* per persoon en € *5.000.000* voor het hele onderzoek (en € *7.500.000* per jaar voor alle onderzoeken van dezelfde opdrachtgever).

Let op: de verzekering dekt de volgende schade **niet**:

- Schade door een risico waarover we u informatie hebben gegeven in deze brief. Maar dit geldt niet als het risico groter bleek te zijn dan we van tevoren dachten. Of als het risico heel onwaarschijnlijk was.
- Schade aan uw gezondheid die ook zou zijn ontstaan als u niet aan het onderzoek had meegedaan.
- Schade die ontstaat doordat u aanwijzingen of instructies niet of niet goed opvolgde.
- Schade aan de gezondheid van uw kinderen of kleinkinderen.
- Schade door een behandelmethode die al bestaat. Of door onderzoek naar een behandelmethode die al bestaat.

Deze bepalingen staan in het 'Besluit verplichte verzekering bij medisch-wetenschappelijk onderzoek met mensen 2015'. Dit besluit staat in de Wettenbank van de overheid (https://wetten.overheid.nl).

Bijlage C: toestemmingsformulier proefpersoon

Behorende bij

Geïndividualiseerde tromboseprofylaxe voor patiënten die een heup of knie vervangende operatie ondergaan: een nationale, multicenter, gerandomiseerde, multi-arm, open label trial.

- Ik heb de informatiebrief gelezen. Ook kon ik vragen stellen. Mijn vragen zijn goed genoeg beantwoord. Ik had genoeg tijd om te beslissen of ik meedoe.
- Ik weet dat meedoen vrijwillig is. Ook weet ik dat ik op ieder moment kan beslissen om toch niet mee te doen met het onderzoek. Of om ermee te stoppen. Ik hoef dan niet te zeggen waarom ik wil stoppen.
- Ik geef de onderzoeker toestemming om mijn huisarts en behandelend specialist te laten weten dat ik meedoe aan dit onderzoek.
- Ik geef de onderzoeker toestemming om informatie op te vragen bij mijn huisarts of specialist(en) die mij behandelt, dit gaat alleen om gegevens relevant voor het onderzoek.
- Ik geef de onderzoeker toestemming om mijn huisarts of specialist informatie te geven over onverwachte bevindingen uit het onderzoek die van belang zijn voor mijn gezondheid.
- Ik geef de onderzoekers toestemming om mijn gegevens te verzamelen en gebruiken. De onderzoekers doen dit alleen om de onderzoeksvraag van dit onderzoek te beantwoorden.
- Ik geef toestemming aan de onderzoekers van het LUMC om mijn contactgegevens te gebruiken voor het versturen van de vragenlijsten, om mij op te bellen als de vragenlijst niet (compleet) is ingevuld, of als er naar aanleiding van de vragenlijst nog onduidelijkheden zijn en om mij te benaderen voor het invullen van een vragenlijst 12 maanden na mijn operatie.
- Ik geef toestemming mijn gegevens over mijn operatie en vragenlijsten die opgeslagen zijn in de database Landelijke registratie orthopedische implantaten (LROI) te gebruiken voor dit onderzoek.
- Ik weet dat voor de controle van het onderzoek sommige mensen al mijn gegevens kunnen inzien. Die mensen staan in deze informatiebrief. Ik geef deze mensen toestemming om mijn gegevens in te zien voor deze controle.
- Wilt u in de tabel hieronder ja of nee aankruisen?

Ik geef toestemming om mij eventueel na dit onderzoek te vragen of ik wil meedoen met een vervolgonderzoek.	Ja ☐	Nee☐
Ik geef toestemming om mijn gegevens te bewaren om deze te gebruiken voor ander onderzoek, zoals in de informatiebrief staat.	Ja ☐	Nee☐

- Ik wil meedoen aan dit onderzoek.

Mijn naam is (proefpersoon): ………………………………..

Handtekening: ……………………… Datum : __ / __ / __

Ik verklaar dat ik deze proefpersoon volledig heb geïnformeerd over het genoemde onderzoek.

Wordt er tijdens het onderzoek informatie bekend die die de toestemming van de proefpersoon kan beïnvloeden? Dan laat ik dit op tijd weten aan deze proefpersoon.

Naam onderzoeker (of diens vertegenwoordiger):……………………………….

Handtekening:……………………… Datum: __ / __ / __

De proefpersoon krijgt een volledige informatiebrief digitaal of op papier, samen met een getekende versie van het toestemmingsformulier.
