# Supplementary material for: Study protocol for the DISTINCT trial: inDividual, targeted thrombosIS prophylaxis versus the standard ‘one-size-fits-all’ approach in patients undergoing Total hIp or total kNee replaCemenT – a national, multicentre, randomised, multiarm, open-label trial
Source: BMJ Open. 2025 Oct 6;15(10):e101180. doi: 10.1136/bmjopen-2025-101180 (PMC12506130; doi:10.1136/bmjopen-2025-101180)
Supplement: Supplementary file 4 [file bmjopen-2025-101180.reviewer_comments.pdf]

PEER REVIEW HISTORY

BMJ Open publishes all reviews undertaken for accepted manuscripts. Reviewers are asked to complete a checklist review form and are provided with free text boxes to elaborate on their assessment. These free text comments are reproduced below.

ARTICLE DETAILS

Title (Provisional)

Study protocol for the DISTINCT trial: inDividual, targeted thrombosIS prophylaxis versus the standard 'one size fits all' approach in patients undergoing Total hIp or total kNee replaCemenT: a national, multicenter, randomized, multi-arm, open-label trial.

Authors

Kok, Ruben Yustian; van Bodegom - Vos, Leti; Ettema, Harmen B; Groenwold, Rolf; van den Hout, Wilbert B; Huisman, Menno V; Klok, Frederikus A; Nelissen, Rob GHH; van Rein, Nienke; van Veen, Merel; Vehmeijer, Stephan BW; Wiegerinck, Jan Joost I; Cannegieter, Suzanne C; Nemeth, Banne

---

VERSION 1 - REVIEW

**Reviewer**	**1**
**Name**	**C Chan , Noel**
**Affiliation**	**Department of Medicine, Division of Hematology and Thromboembolism, McMaster University**
**Date**	**03-Aug-2025**
**COI**	**None**

In this manuscript, Kok R and colleagues present the design and rationale of the DISTINCT trial - a national, multicenter, randomized, multi-arm, open-label study evaluating the efficacy and safety of an individualized thromboprophylaxis strategy in patients undergoing total hip or knee arthroplasty (THA/TKA). The trial utilizes the validated TRiP(plasty) score to stratify patients into low, intermediate, and high venous thromboembolism (VTE) risk categories, with tailored interventions aimed at de-escalating prophylaxis in low-risk patients and escalating it in high-risk patients.

DISTINCT will be the first large-scale, adequately powered trial to personalize thromboprophylaxis based on a risk score, and its findings have the potential to significantly influence clinical practice. The manuscript is well written and the study has been thoughtfully conceived. I have a few suggestions and comments outlined below:

Comments/Suggestions:

1) For patients in the intermediate risk group (DISTINCT-2), is there scope to further simplify therapy—particularly if the expected VTE risk falls between 1.0–1.5%? The EPCAT trials suggest that anticoagulant prophylaxis could be shortened or transitioned to antiplatelet

therapy upon discharge. Some clarification on whether this ought to be considered would be valuable.

2) Consistency regarding risk threshold: The manuscript states that for DISTINCT-2, the 95% confidence interval (CI) is expected to range from 0.9% to 1.7%, with less than a 15% chance that the upper bound will exceed the 2% margin. To align with the TRiP(plasty) risk stratification framework, should the target upper bound not be <1.5%? Similarly, for DISTINCT-1, should the non-inferiority margin be <1% to remain consistent with the low-risk classification.

3) Regarding sample size assumptions for DISTINCT 3, the sample size calculation for DISTINCT-3 assumes a 50% relative risk reduction. Data from the pooled analysis of the ADVANCE trials suggest a lower bound of approximately 30%, which would still be clinically meaningful. Would the current sample size be adequately powered to detect a 30% reduction in VTE in the intervention arm?

Minor comments:

4) It would be useful to include the anticipated distribution of patients across the low, intermediate, and high VTE risk groups—either in the text or in a figure. Historical data would be useful to support the feasibility of recruiting these numbers during the expected trial duration in the Netherlands.

5) In Figure 1, consider adding the TRiP(plasty) score threshold for each stratum of risk for clarity.

**Reviewer**	**2**
**Name**	**Harris, Ian A.**
**Affiliation**	**University of New South Wales South Western Sydney Clinical School**
**Date**	**05-Aug-2025**
**COI**	**None**

This manuscript reports the protocol for a study comparing targeted VTE prophylaxis to standard VTE prophylaxis for hip and knee replacement surgery. This area remains controversial so further evidence from high quality trials is welcome. Comments are as follows:

1. Routine or low-threshold screening for VTE may complicate the outcome measurement. Some clarity around protocols avoiding routine screening should be mentioned.

2. The abbreviation "DISTINCT" has already been used for another arthroplasty trial, which might be confusing (Farey et al, BMJ Open 2022: Dual mobility versus conventional total hip arthroplasty in femoral neck fractures (DISTINCT): protocol for a registry-nested, open-label, cluster-randomised crossover trial)

3. Suggest adding “within 90 days” after “VTE risk” to study aims, just to make it much clearer.

4. The “3 month” and “90 day” periods seem to be used interchangeably. I suggest picking one and sticking with it.

5. The primary outcome (VTE) appears to be detected by patient reported questionnaires. IT is stated that “additional data” will be acquired for those who report VTE – I assume this extra data is something like “confirmation of VTE status from imaging studies”. If so, it is better to report that specifically.

6. Is there any screening for false negatives (a patient who had a VTE but failed to report it in the questionnaire?

7. The non-inferiority margin of 1% for DISTINCT 1 is very large, and much higher than the expected incidence (0.75%). I find it hard to believe that surgeons would accept a VTE rate of 1.75% compared to 0.75%. Unless, by a “non-inferiority limit” the authors mean that they will accept a VTE rate of up to 1% (compared to 0.75% in the control group), which sounds reasonable. This needs to be clarified: is the non-inferiority margin 1% (meaning a difference between groups of up to 1%) or does the term “non-inferiority limit” (a term unknown to me) of 1% mean that the expect VTE rate in the intervention group is up to 1% (rather than 1.75%)?

VERSION 1 - AUTHOR RESPONSE

Reviewer: 1

Dr. Noel C Chan , Department of Medicine, Division of Hematology and Thromboembolism, McMaster University

Comments to the Author:

In this manuscript, Kok R and colleagues present the design and rationale of the DISTINCT trial - a national, multicenter, randomized, multi-arm, open-label study evaluating the efficacy and safety of an individualized thromboprophylaxis strategy in patients undergoing total hip or knee arthroplasty (THA/TKA). The trial utilizes the validated TRiP(plasty) score to stratify patients into low, intermediate, and high venous thromboembolism (VTE) risk categories, with tailored interventions aimed at de-escalating prophylaxis in low-risk patients and escalating it in high-

risk patients.

DISTINCT will be the first large-scale, adequately powered trial to personalize thromboprophylaxis based on a risk score, and its findings have the potential to significantly influence clinical practice. The manuscript is well written and the study has been thoughtfully conceived. I have a few suggestions and comments outlined below:

Comments/Suggestions:

1) For patients in the intermediate risk group (DISTINCT-2), is there scope to further simplify therapy—particularly if the expected VTE risk falls between 1.0–1.5%? The EPCAT trials suggest that anticoagulant prophylaxis could be shortened or transitioned to antiplatelet therapy upon discharge. Some clarification on whether this ought to be considered would be valuable.

In the current trial, the inclusion of DISTINCT-2 mainly serves as a safety margin, as the precise cutoff between low- and high VTE risk patients predicted by the TRiP(plasty) score has not yet been established. Once the study is completed, the collected data will be used to refine the optimal cut-off and thromboprophylaxis therapy by weighing the risks and benefits of different

thromboprophylaxis regimens. This could imply that therapy for the intermediate group is further simplified as suggested by the reviewer. At this moment we would like to refrain from

speculations on thromboprophylaxis therapy for the intermediate group as we do not test an intervention for this group of patients.

2) Consistency regarding risk threshold: The manuscript states that for DISTINCT-2, the 95% confidence interval (CI) is expected to range from 0.9% to 1.7%, with less than a 15% chance that the upper bound will exceed the 2% margin. To align with the TRiP(plasty) risk stratification framework, should the target upper bound not be <1.5%? Similarly, for DISTINCT-1, should the non-inferiority margin be <1% to remain consistent with the low-risk classification.

We apologise this may have been unclear. The expected point estimate (absolute S0-day VTE risk) for DISTINCT-2 is 1.3%, this serves as the primary result of this study arm. To achieve a confidence interval with an upper bound below 1.5%, a very large sample size would be

required, making feasibility questionable. Moreover, since patients with an estimated risk between 1.0% and 1.5% are included, the true point estimate could theoretically be as high as

1.4S%. In such a case, obtaining a S5% CI with an upper bound below 1.5% would be virtually impossible. Taking this into account, together with the objectives of DISTINCT-2, its role as a safety margin and its contribution to improving the TRiP(plasty) model. We consider the S5% CI specified in the protocol to provide sufficient precision.

Regarding the non-inferiority margin of DISTINCT-1, choosing a non-inferiority margin is a trade- off between trial feasibility and clinical judgement (weighing the risks and benefits of all primary and secondary outcomes). For our trial, the non-inferiority margin was based on clinician

opinion and two recent randomised controlled trials on the same topic which used the same margin (N Engl J Med 2018;378:cSS-707 and JAMA 2022;328;(8):71S-727.) We believe this supports our non-inferiority margin, which is consistent with the minimally clinically important difference of 1% described in the literature.

3) Regarding sample size assumptions for DISTINCT 3, the sample size calculation for DISTINCT- 3 assumes a 50% relative risk reduction. Data from the pooled analysis of the ADVANCE trials suggest a lower bound of approximately 30%, which would still be clinically meaningful. Would the current sample size be adequately powered to detect a 30% reduction in VTE in the intervention arm?

DISTINCT-3 was powered to detect a 50% relative risk reduction in symptomatic VTE. Unfortunately, the study is not adequately powered to accommodate the detection of a 30% risk reduction (as suggested by the lower bound of the CI) .

However, it is important to note that the apixaban dosage used in the ADVANCE trials was 2.5 mg twice daily, whereas in DISTINCT-3 patients receive 5 mg twice daily, with a comparable control regimen. Based on this higher dosing, we expect a risk reduction at least comparable to, if not greater than, the absolute risk difference of 0.45% observed in the ADVANCE pooled analysis.

This expectation is further supported by the APROPOS dose-finding trial (Lassen et al., 2007), in which patients were randomized to different dosage of apixaban thromboprophylaxis after TKA. That study showed a dose-dependent reduction in VTE incidence. Specifically, the 2.5 mg b.i.d. and 5 mg b.i.d. regimens resulted in a VTE incidence of 10/111 and 5/105, respectively, corresponding to a relative risk of 0.53 (S5% CI: 0.1S–1.50).

Additionally, in a pilot study conducted in preparation for DISTINCT, we compared postoperative VTE incidence in patients with atrial fibrillation receiving therapeutic DOACs (comparable to the DISTINCT-3 intervention) to those receiving standard thromboprophylaxis after THA/TKA. This showed a relative risk of 0.c5. However, this likely underestimates the true treatment effect, as no adjusted analyses were performed and patients with atrial fibrillation typically have more comorbidities than the general THA/TKA population. (J Thromb Thrombolysis. 2024 Oct;57(7):124S-1255)

Based on these studies a risk reduction of 50% is to be expected. Moreover, powering DISTINCT- 3 to detect a 30% risk would require a sample size of approximately 13,000 patients. Given that only about 15% of the THA/TKA population is expected to be classified as high risk, such a requirement would pose serious challenges to the feasibility of the trial.

Minor comments:

4) It would be useful to include the anticipated distribution of patients across the low, intermediate, and high VTE risk groups—either in the text or in a figure. Historical data would be useful to support the feasibility of recruiting these numbers during the expected trial duration in the Netherlands.

We agree this to be informative and have added the following information on the expected distribution and feasibility of the trial under patient screening and recruitment (lines 1c2-1c5): “Based on the validation study of the TRiP(plasty) score, the expected distribution of patients over DISTINCT 1, DISTINCT 2 and DISTINCT 3 is 55%, 30% and 15% respectively.[18] We aim to include a minimum of 10 participating centers, which collectively perform approximately 17,150 THA/TKA procedures annually, ensuring the feasibility of the study.”

5) In Figure 1, consider adding the TRiP(plasty) score threshold for each stratum of risk for clarity.

We added the score threshold for DISTINCT-2 (1.0%-1.5%) in figure 1.

Reviewer: 2

Dr. Ian A. Harris, University of New South Wales South Western Sydney Clinical School Comments to the Author:

This manuscript reports the protocol for a study comparing targeted VTE prophylaxis to standard VTE prophylaxis for hip and knee replacement surgery. This area remains controversial so further evidence from high quality trials is welcome. Comments are as follows:

1. Routine or low-threshold screening for VTE may complicate the outcome measurement. Some clarity around protocols avoiding routine screening should be mentioned.

Except for the thromboprophylaxis protocol, all study patients will receive standard postoperative care, which does not involve routine VTE screening, testing for VTE will only be performed in cases of clinical suspicion. No specific measures have been implemented to prevent potential increases in VTE testing due to a lower threshold among healthcare providers.

However, in our previous POT-CAST and POT-KAST trials with a comparable open-label design (thromboprophylaxis vs. no thromboprophylaxis after knee arthroscopy or lower-leg cast), we observed that the diagnosis VTE was confirmed in a comparable rate in both treatment groups indicating no major shift in the testing threshold (van Adrichem et al., NEJM 2017). We have clarified this under Study follow-up (lines 255-25c): "All postoperative care is according to the current standard of care, no routine VTE screening will be performed."

2. The abbreviation "DISTINCT" has already been used for another arthroplasty trial, which might be confusing (Farey et al, BMJ Open 2022: Dual mobility versus conventional total hip arthroplasty in femoral neck fractures (DISTINCT): protocol for a registry-nested, open-label, cluster-randomised crossover trial)

We agree this might be confusing and apologize we missed this. The first project idea including trial name was submitted in November 2021, we checked most trial registries, unfortunately we did not check the ANZCTR. At this stage, changing the study acronym is no longer feasible, as

the study has already begun enrolling participants.

3. Suggest adding "within 90 days" after "VTE risk" to study aims, just to make it much clearer.

We added within S0 days postoperative after VTE risk in our study objectives.

4. The "3 month" and "90 day" periods seem to be used interchangeably. I suggest picking one and sticking with it.

We agree with this suggestion: where 3 month was used we changed it to S0 day throughout the manuscript.

5. The primary outcome (VTE) appears to be detected by patient reported questionnaires. IT is stated that "additional data" will be acquired for those who report VTE – I assume this extra data is something like "confirmation of VTE status from imaging studies". If so, it is better to report that specifically.

If a participant reports a (possible) outcome event all relevant medical data will be collected. This includes: discharge letters, status reports, laboratory results, results from medical microbiology, radiology results, nuclear medicine results, autopsy reports, or any other relevant document. We added clarification under study follow-up (lines 25c-258): "In case of a (possible) outcome event, additional data on the event including imaging studies, consult letters,

discharge letters and any other relevant information are collected from the medical record of the participant."

6. Is there any screening for false negatives (a patient who had a VTE but failed to report it in the questionnaire?

If a outcome event is observed by a member of the study team in a participating centre, this will be reported to the coordinating centre. There is no screening for false negatives. However we do ask if any additional patient visits or imaging has taken place. If patients are lost to follow-up

their general practitioner will be approached to not mis any outcome events.

7. The non-inferiority margin of 1% for DISTINCT 1 is very large, and much higher than the expected incidence (0.75%). I find it hard to believe that surgeons would accept a VTE rate of 1.75% compared to 0.75%. Unless, by a "non-inferiority limit" the authors mean that they will accept a VTE rate of up to 1% (compared to 0.75% in the control group), which sounds
reasonable. This needs to be clarified: is the non-inferiority margin 1% (meaning a difference between groups of up to 1%) or does the term "non-inferiority limit" (a term unknown to me) of 1% mean that the expect VTE rate in the intervention group is up to 1% (rather than 1.75%)?

The non-inferiority limit is equivalent to the non-inferiority margin, meaning we will accept an **upper bound** of the one-sided S7.5% confidence interval up to 1.75%. This was based on clinician opinion and two recent randomised controlled trials on the same topic which used the same margin (N Engl J Med 2018;378:cSS-707 and JAMA 2022;328;(8):71S-727.)

It is important to consider that our goal is to balance thrombosis risk and bleeding risk. Reducing the intensity of thromboprophylaxis is expected to lower the incidence of CRNMB and minor bleeding, which may also reduce the risk of PJI. This rationale supports the choice of a larger non-inferiority margin, consistent with the minimally clinically important difference of 1%.
